# The FRiND Model: A Mathematical Model for Representing Macrophage Plasticity in Muscular Dystrophy Pathogenesis

**DOI:** 10.1007/s11538-019-00635-8

**Published:** 2019-07-13

**Authors:** Matthew T. Houston, Juan B. Gutierrez

**Affiliations:** 1 Department of Mathematics, Middle Georgia State University, Macon, GA 31206 USA; 2 University of Georgia, Athens, GA 30602 USA; 3 University of Texas, San Antonio, San Antonio, TX 78249 USA

**Keywords:** Systems biology, Muscular dystrophy, Mathematical models, MD, LGMD, Ordinary differential equations, Muscle repair, Immunology, 92C42, 92D25, 65L06, 34-04

## Abstract

Muscular dystrophy describes generalized progressive muscular weakness due to the wasting of muscle fibers. The progression of the disease is affected by known immunological and mechanical factors, and possibly other unknown mechanisms. This article introduces a new mathematical model, the FRiND model, to further elucidate these known immunological actions. We will perform stability and sensitivity analyses on this model. The models time course results will be verified by biological studies in the literature. This model could be the foundation for further understanding of immunological muscle repair.

## Introduction

Muscular dystrophy (MD) is a group of genetic disorders whose archetypal pathology is progressive weakening of skeletal muscle tissue. Severity of degeneration and muscles affected vary depending on the type of MD (and person) with some forms leading to early death while other forms remain unnoticed until adulthood. All forms of MD combined affect about 37 per 100, 000 individuals in Northern England (Norwood 2009). Duchenne’s muscular dystrophy (DMD)is the most prevalent of all childhood genetic disorders affecting males (Norwood 2009).

Muscle degeneration in MD can be caused by replacement of healthy muscle fiber by fibrous connective (Dreyfus et al. 1954) and adipose tissue (Pichiecchio et al. 2002); tissue replacement could be caused by chronic inflammation and immune activation. Chronic inflammation is induced by increased immune activity, with a higher likelihood of cellular damage as a consequence of weaknesses in cell structure proteins—caused by MD affected genes (Wehling et al. 2001; Selva-O’Callaghan et al. 2006).

Macrophages, a type of innate immune cell, perform a significant role in tissue repair (Arnold et al. 2007). Macrophages phagocytose damaged muscle fibers (Arnold et al. 2007) and promote the proliferation, differentiation, and binding of myoblasts into nascent myofibers which form new muscle tissue (Ogawa et al. 2015). Macrophages accomplish these tasks by switching phenotype (macrophage plasticity) throughout the repair process. While in an inflammatory phenotype, macrophages migrate to damaged tissue, perform phagocytosis, and promote myoblast proliferation. Macrophages follow this stage with anti-inflammatory phenotypes which phagocytose, halt inflammation, differentiate myoblasts, lay fibrous connective tissue which gives structure to muscle, and signal myotube migration/binding.

Although many mathematical models have been created to study the immune defense, few have been devised to quantify tissue regeneration by the immune system and chronic inflammation (Houston et al. 2018). Dell’Acqua and Castiglione (2009) and Jarrah et al. (2014) both attempt to explain muscle degeneration and regeneration by the interactions of macrophages, T helper cells, and cytotoxic T cells.

Both previous models fail to elaborate on macrophage plasticity and the relation between acute and chronic inflammation. The previous models treated all macrophages as a single group that acts on all other cells the same. However, Arnold et al. (2007) showed that macrophages have several phenotypes that interact with other cells differently. The previous models also investigated the long-term effects a single acute, damage event. The weakening of skeletal muscle tissue in MD, though, occurs after continuous damage from everyday activity which causes a chronic inflammation (Weller et al. 1990).

The purpose of this article is to present a mathematical model that simulates macrophage plasticity and its effects on muscle regeneration. A correct characterization of this dynamics could allow a greater understanding of the causes of chronic inflammation, and muscle degeneration in MD patients.

## The FRiND Model

### Concept of the FRiND Model

**Fig. 1:**
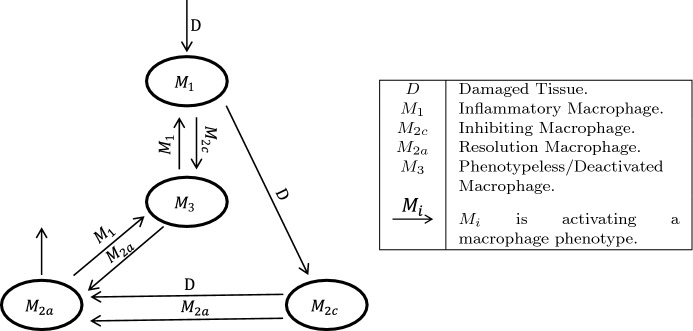
*Macrophage interactions* This figure describes the interaction of different types of macrophages during muscle repair. Inflammatory signals from resident macrophages are released after tissue is damaged prompting invasion by pro-inflammatory macrophages ($M_1$). Phagocytosis of damaged tissue by pro-inflammatory macrophages promotes inhibiting macrophage phenotype ($M_{2c}$) which starts deactivating ($M_3$) pro-inflammatory macrophages. Resolution macrophages ($M_{2a}$) appear after further phagocytosis of damaged tissue by inhibiting macrophages. $M_{2a}$ macrophages slowly apoptose during muscle repair and following resolution of repair will return to starting population of resident macrophages. Conflicting signals by pro- and anti-inflammatory macrophages force some macrophages into a phenotypeless state ($M_3$)

**Fig. 2:**
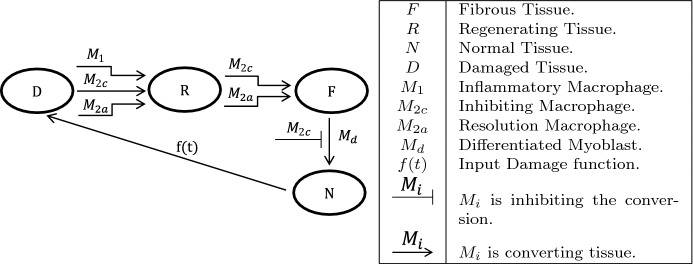
*Muscle tissue repair* This figure shows the process of muscle repair. Phagocytosis of damaged tissue (*D*) by macrophages gives regenerating tissue (*R*). Fibrous connective tissue (*F*) is laid by some types of macrophages over regenerating tissue. Fibrous tissue binds with differentiated myoblasts to form normal muscle tissue (*N*)

**Fig. 3:**
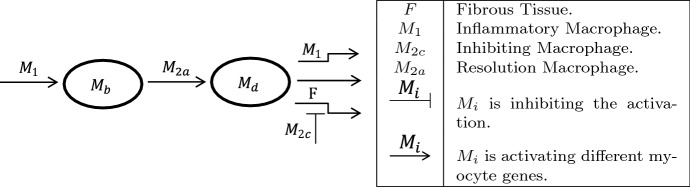
*Formation of nascent myofibers* This figure shows the creation of nascent myofibers. Pro-inflammatory macrophages promote activation and proliferation of myoblasts ($M_b$). Exposure to resolution macrophages promotes differentiation of myoblasts ($M_d$) which eventually bind together into myofibers

To understand the interactions needed for muscle repair, we could create a model that incorporates three groups of cells: macrophages (in number of cells per $\mathrm{mm}^3$), muscle tissue cells (in percentage of tissue), and myocytes (in number of cells per $\mathrm{mm}^3$). The interactions of these cells in a mathematical model should ideally stay in equilibrium until any damage to muscle occurs (Figs. 1,2,3).

For purposes of this model, macrophage populations have been split into four phenotypes derived from in vitro studies: $$M_1,$$$$M_{2c},$$$$M_{2a},$$ and $$M_3$$. Although in vivo studies have shown few macrophages display the exact qualities of these phenotypes, they can be shown to have certain genes—corresponding to those in vitro phenotypes—upregulated (Novak et al. 2014).

**Since monocytes infiltrate into damage tissue as** pro-inflammatory macrophages ($$M_1$$,) the $$M_1$$ population increases proportionally to the damage dealt by injury. $$M_1$$ macrophages release cytokines $$TNF_{\alpha }$$ and IL-1, which further allow $$M_1$$ infiltration and recruitment of phenotypeless macrophages $$(M_3); $$**this is modeled using**$$k_1D^2$$. These cytokines also promote pro-inflammatory genes in resident macrophages forcing them into an $$M_3$$ phenotypeless state. TGF-$$\beta $$ is released when macrophages phagocytose damaged tissue and apoptotic immune cells; this promotes a shift in macrophages to an anti-inflammatory phenotype (Arnold et al. 2007).

Anti-inflammatory macrophages ($M_2$) fall into two phenotypes: the deactivating macrophages ($M_{2c}$) and resolving macrophages ($M_{2a}$). Increased exposure to TGF-$\beta$ and certain glucocorticoids promote $M_{2c}$ phenotype genes in $M_1$ macrophages. IL-10 which is released by $M_{2c}$ macrophages inhibits the effects of IL-1 and suppresses $M_1$ macrophage’s inflammatory abilities effectively becoming phenotyp**e**less. Further exposure by $M_{2c}$ to TGF-$\beta$ promotes a shift to the $M_{2a}$ phenotype. $M_{2a}$ macrophages release cytokines IL-4 and IL-13 which accelerate $M_{2c}$ phenotype change and promote myoblasts differentiation (Zhang et al. 2016).

Phenotypeless **or “switch”** macrophages ($M_3$) are used in this model to represent any macrophage which has a phenotype not sufficiently polarized or is suppressed by another macrophage. The $M_3$ macrophages will act only as a feedback loop for $M_1$ and $M_{2a}$ (Malyshev and Malyshev 2015; Kalish et al. 2017).

Following damage to normal muscle tissue (*N*), damage tissue (*D*) increases. Macrophages phagocytose this tissue and leave the area ready for regeneration (*R*). Transforming Growth Factor Beta, TGF-$\beta ,$ which is released by $M_2$ macrophages promotes the formation of fibrous tissue (*F*) (Arnold et al. 2007; Mounier et al. 2013). This tissue is the foundation upon which myotubes bind to form new healthy muscle tissue (*N*) (Ogawa et al. 2015). In the model, the *F*,  *R*,  *N*,  and *D* are given as percents of overall tissue as this is assumed to be conserved.

A subpopulation of satellite cells called myocytes form the core of new muscles. Pro-inflammatory signals from $M_1$ macrophages activate *MyoD* in resident satellite cells becoming myoblasts allowing for proliferation. The model simplifies this by combining the resident satellite cells and myoblasts into $M_b$. When exposed to anti-inflammatory signals by $M_{2a},$ myoblasts lose the expression of *Pax* and differentiate. These differentiated myoblasts later form into myotubes. The model combines the differentiated myoblasts and myotubes as a single variable, $M_d$. $M_1$ macrophages are known to signal $M_d$ myoblasts to prematurely apoptose (Ogawa et al. 2015).

Damage to muscle tissue is controlled by a time-dependent function *f*(*t*) which can be designed to simulate different types of muscle injury. For acute injury, *f*(*t*) could be set as a Gaussian normal or a **Gaussian** function as described by Jarrah et al. (2014). For smaller but periodic injury, *f*(*t*) could be set with a random amplitude and period $\sin ^2$ function. **This last function will be discussed in** Sect. 3.3.

We will call this model the FRiND (*F*ibrous, *R*egenerating, inflammation, *N*ormal, *D*amaged) model.

### Equations

The FRiND model uses the law of generalized mass action (GMA) to convert the diagrams in Sect. 2.1 into ordinary differential equations (ODEs). GMA states that the rate of reaction/interaction is directly and/or indirectly proportional to the product of the numbers (or masses) of objections. For each interaction, a kinetic parameter is incorporated and is estimated using data. In the FRiND model, activation and deactivation will be modeled using direct proportions; inhibition of an interaction will be modeled using an indirect proportion. An extra term $- g(t)M_1$ has been added for predictions of Diphtheria toxin in Sect.  3.2; we assume $g(t) = 0$ in **all** other section**s**.

**FRiND Model**

1 $$\begin{aligned} \dot{F}&= d_1M_{2c}R + d_2M_{2a}R - \dfrac{d_6M_dF}{M_{2c}-M_{2c}(0) + 1}, \end{aligned}$$

2 $$\begin{aligned} \dot{R}&= d_3M_1D - d_1M_{2c}R + d_4M_{2a}D + d_5M_{2c}D - d_2M_{2a}R, \end{aligned}$$

3 $$\begin{aligned} \dot{N}&= \dfrac{d_6M_dF}{M_{2c}-M_{2c}(0) + 1} - f(t)N, \end{aligned}$$

4 $$\begin{aligned} \dot{D}&= f(t)N - d_3M_1D - d_4M_{2a}D - d_5M_{2c}D, \end{aligned}$$

5 $$\begin{aligned} \dot{M_1}&= k_1D^2 - k_2M_1D - k_3M_1M_{2c} + k_4M_1M_3 - g(t)M_1, \end{aligned}$$

6 $$\begin{aligned} \dot{M_{2c}}&= k_2M_1D - k_5\left( M_{2c} - M_{2c}(0)\right) D - k_8\left( M_{2c}-M_{2c}(0)\right) M_{2a}, \end{aligned}$$

7 $$\begin{aligned} \dot{M_{2a}}&= k_5\left( M_{2c}-M_{2c}(0)\right) D + k_6M_{2a}M_3 - k_7M_1M_{2a},\nonumber \\&\quad + k_8\left( M_{2c}-M_{2c}(0)\right) M_{2a} - k_9\left( M_{2a} - M_{2a}(0)\right) , \end{aligned}$$

8 $$\begin{aligned} \dot{M_3}&= k_3M_1M_{2c} + k_7M_1M_{2a} - k_4M_1M_3 - k_6M_{2a}M_3, \end{aligned}$$

9 $$\begin{aligned} \dot{M_b}&= a_1M_1M_b - a_2\left( M_b - M_b(0) \right) M_{2a}, \end{aligned}$$

10 $$\begin{aligned} \dot{M_d}&= a_2\left( M_b - M_b(0) \right) M_{2a} - a_4M_d - a_5M_1M_d - \dfrac{a_3M_dF}{M_{2c}-M_{2c}(0) + 1}. \end{aligned}$$

**Table:** 

Variable	Description	Variable	Description
*F*	Fibrous tissue	$M_1$	Macrophage 1 (inflammatory)
*R*	Regenerating tissue	$M_{2c}$	Macrophage 2c (deactivating)
*N*	Normal tissue	$M_{2a}$	Macrophage 2a (resolving)
*D*	Damaged tissue	$M_3$	No phenotype macrophage
$M_b$	Myoblasts	$M_d$	Differentiated myotubes

### Estimating the Model Parameters

The FRiND Model has 20 parameters plus any parameters needed for the initial damage function.

Data for estimating the parameters come from Mounier et al. (2013) and Ogawa et al. (2015) (see Table 1). The choice of these literature sources was influenced by the similarity of the data collected with respect to the model proposed here; other sources include the adaptive immune system. Both experiments used cardiotoxin (CTX) as a source of damage to mouse muscle and similar control mice. The damage by CTX is modeled using a **Gaussian function**:

11 $$\begin{aligned} f(t) = \dfrac{h}{\sigma \sqrt{2 \pi } }\exp {\left( -\dfrac{1}{2 \sigma ^2}\left( t - m \right) ^2\right) }. \end{aligned}$$

A Gaussian function was chosen to model CTX to allow for a symmetric growth and decay with a predictable peak within one day that causes damaged tissue to reach over 80% (Mounier et al. 2013; Hardy et al. 2016). However, nearly any bell-shaped or log-bell function in time works when applied to the FRiND model.

The data were collected from a small number of figures pertaining to the control mice in each paper. $M_3$ macrophage numbers were developed by subtracting the total macrophage populations by the number of $M_1$ and $M_2$ macrophages. To make estimating parameters easier, interpolation was used to calculate myocyte data on days when tissue data were present. The data for days 14 and 24 were the same as initial conditions since both papers report resolution of repair within 14 days.

**Table 1:** *Data used to estimate parameters* This table gives the data used to estimate the parameters for the FRiND model. The data were gleamed from Mounier et al. (2013) and Ogawa et al. (2015)

*Time*	*F*	*R*	*N*	*D*	$M_1$	$M_2$	$M_3$	$M_b$	$M_d$
0	0	0	100	0	0	400	0	100	0
2	0	45	0	55	5000	5000	2500	1000	0
4	70	25	0	1	1000	5000	1500	1412	1427
7	5	2	95	0	250	2,000	375	500	100
14	0	0	100	0	0	400	0	100	0
24	0	0	100	0	0	400	0	100	0

A standard genetic algorithm (Bäck et al. 1997) and pattern search (Hooke and Jeeves 1961) were used to train parameters to the data on both COPASI (Hoops et al. 2006) and MATLAB (The MathWorks, Inc., Natick, MA, USA).

**Table 2:** *Estimated parameters for the FRiND model* This table shows the estimated parameters for the FRiND model using an acute *Gaussian* damage with data from 1

Parameter name	Estimated value	Units	Parameter name	Estimated value	Units
$$a_1$$	0.0003420387898	$$\dfrac{\mathrm{mm}^3}{\mathrm{cells}\, \mathrm{day}}$$	$$k_1 $$	0.9167670584	$$\dfrac{\mathrm{mm}^3}{\mathrm{cells}\, \mathrm{day}}$$
$a_2$	0.00027805269	$\dfrac{\mathrm{mm}^3}{\mathrm{cells}\, \mathrm{day}}$	$k_2$	0.007512675612	$\dfrac{\mathrm{mm}^3}{\mathrm{cells}\, \mathrm{day}}$
$a_3$	5.926671787	$\dfrac{1}{\mathrm{day}}$	$k_3$	0.01113163887	$\dfrac{\mathrm{mm}^3}{\mathrm{cells}\, \mathrm{day}}$
$a_4$	0.1685466672	$\dfrac{1}{\mathrm{day}}$	$k_4$	0.01332585023	$\dfrac{\mathrm{mm}^3}{\mathrm{cells}\, \mathrm{day}}$
$a_5$	0.0004735032862	$\dfrac{\mathrm{mm}^3}{\mathrm{cells}\, \mathrm{day}}$	$k_5$	0.002183270219	$\dfrac{\mathrm{mm}^3}{\mathrm{cells}\, \mathrm{day}}$
$d_1$	0.0001	$\dfrac{\mathrm{mm}^3}{\mathrm{cells}\, \mathrm{day}}$	$k_6$	0.001135187023	$\dfrac{\mathrm{mm}^3}{\mathrm{cells}\, \mathrm{day}}$
$d_2$	0.000199043595	$\dfrac{\mathrm{mm}^3}{\mathrm{cells}\, \mathrm{day}}$	$k_7$	0.0005064492007	$\dfrac{\mathrm{mm}^3}{\mathrm{cells}\, \mathrm{day}}$
$d_3$	0.00001041101141	$\dfrac{\mathrm{mm}^3}{\mathrm{cells}\, \mathrm{day}}$	$k_8$	0.0002272581636	$\dfrac{\mathrm{mm}^3}{\mathrm{cells}\, \mathrm{day}}$
$d_4$	0.0005912309411	$\dfrac{\mathrm{mm}^3}{\mathrm{cells}\, \mathrm{day}}$	$k_9$	0.6179192106	$\dfrac{1}{\mathrm{day}}$
$d_5$	0.000103662336	$\dfrac{\mathrm{mm}^3}{\mathrm{cells}\, \mathrm{day}}$	*h*	9.466706714	$\dfrac{1}{\mathrm{day}}$
$d_6$	0.310343	$\dfrac{1}{\mathrm{day}}$	*m*	0.2	$\ln {(\mathrm{days})}$
$\sigma$	0.01				

The parameters $d_3,\ d_4, \text{ and } d_5$ are misleading when first observed. These values represent $M_1,\ M_{2c}, \text{ and } M_{2a}$ macrophages, respectively, ability to perform phagocytosis not the cytotoxicity. In Mounier et al. (2013), they demonstrated that in AMPK$\alpha 1-/-$, mice phagocytosis of damaged tissue was hampered, and $M_1$ macrophages did not transition to $M_2$ macrophages. This leaves the possibility that $M_2$ macrophages had a greater capacity to phagocytose. This has also been speculated in phagocytosis of tumor cells by $M_{2c}$ (Herter et al. 2014) and phagocytosis by $M_{2a}$ in neurological repair (Ghosh et al. 2016). Multiple tries were made to estimate the parameters with $d_3 > d_4$, and all failed to find a satisfactorily low objective value.

### Fixed Points

A steady state is a **multi-dimensional point** where the system will remain constant over time. This occurs when the **point**$\tilde{x}$ (also called a fixed point) gives $\dfrac{\mathrm{d}x}{\mathrm{d}t} = 0$ for all *x*. For a large dynamical system, finding a fixed point analytically can be difficult (Strogatz 2018).

In the FRiND model, several simplifying assumptions can be made. (1) Since the change in substrate amount is given in the model, parameters may be assumed to be always positive and nonzero. Furthermore, (2) steady state and initial conditions of the substrate must be nonnegative to make biological sense. (3) We will, though, insist that $M_b(0)>0$ (the initial condition of $M_b$ myocytes) as this will allow satellite cells to proliferate. Finally, (4) we want to see how the system responds after contractual damage has dissipated or any diphtheria toxins have left; this would require $\lim \limits _{t\rightarrow \infty }{f(t)} = 0$ and $\lim \limits _{t\rightarrow \infty }{g(t)} = 0.$

Although the initial conditions for substrates depend on the situation being modeled, the muscle cells have a built-in conservation law $\dfrac{\mathrm{d}F}{\mathrm{d}t} + \dfrac{\mathrm{d}R}{\mathrm{d}t} + \dfrac{\mathrm{d}N}{\mathrm{d}t} + \dfrac{\mathrm{d}D}{\mathrm{d}t} = 0.$ Since muscle cells are in percentage of muscle tissue, we will have that $F(t) + R(t) + N(t) + D(t) = 100.$

#### Theorem 1

Assume the FRiND model follows the above assumptions and the parameters given in Table 2, then the model will have these two steady states.

$$\begin{aligned} \tilde{x}_1&= \left[ \begin{array}{c} \tilde{F} \\ 0 \\ 100-\tilde{F} \\ 0 \\ 0 \\ M_{2c}(0) \\ M_{2a}(0) \\ 0 \\ M_b(0) \\ 0 \end{array} \right]&\tilde{x}_2 = \left[ \begin{array}{c} 0, \\ 0, \\ 100, \\ 0, \\ \dfrac{k_3k_6M_{2c}(0)}{k_4k_7}, \\ M_{2a}(0), \\ M_{2c}(0), \\ \dfrac{k_3M_{2c}(0)}{k_4}, \\ \dfrac{a_2k_4k_7M_b(0)M_{2a}(0)}{a_2k_4k_7M_{2a}(0) - a_1k_3k_6M_{2c}(0)}, \\ \dfrac{a_1a_2k_3k_4k_6k_7M_{2c}(0)M_{2a}(0)M_b(0)}{\Big (a_2k_4k_7M_{2a}(0) - a_1k_3k_6M_{2c}(0)\Big )\Big (a_4k_4k_7 + a_5k_3k_6M_{2c}(0) \Big )} \end{array} \right] . \end{aligned}$$

**where**$$\tilde{x} = [\tilde{F},\tilde{R},\tilde{N},\tilde{D},\tilde{M}_1,\tilde{M}_{2c},\tilde{M}_{2a},\tilde{M}_3,\tilde{M}_b,\tilde{M}_d]^T$$.

#### Proof

Using the definition of fixed points, we have:

12 $$\begin{aligned} \tiny 0&= d_1\tilde{M}_{2c}\tilde{R} + d_2\tilde{M}_{2a}\tilde{R} - \dfrac{d_6\tilde{M}_d\tilde{F}}{\tilde{M}_{2c}-M_{2c}(0) + 1}, \end{aligned}$$

13 $$\begin{aligned} 0&= d_3\tilde{M}_1\tilde{D} - d_1\tilde{M}_{2c}\tilde{R} + d_4\tilde{M}_{2a}\tilde{D} + d_5\tilde{M}_{2c}\tilde{D} - d_2\tilde{M}_{2a}\tilde{R}, \end{aligned}$$

14 $$\begin{aligned} 0&= \dfrac{d_6\tilde{M}_d\tilde{F}}{\tilde{M}_{2c}-M_{2c}(0) + 1} - f(t)\tilde{N}, \end{aligned}$$

15 $$\begin{aligned} 0&= f(t)\tilde{N} - d_3\tilde{M}_1\tilde{D} - d_4\tilde{M}_{2a}\tilde{D} - d_5\tilde{M}_{2c}\tilde{D}, \end{aligned}$$

16 $$\begin{aligned} 0&= k_1\tilde{D}^2 - k_2\tilde{M}_1\tilde{D} - k_3\tilde{M}_1\tilde{M}_{2c} + k_4\tilde{M}_1\tilde{M}_3 - g(t)\tilde{M}_1, \end{aligned}$$

17 $$\begin{aligned} 0&= k_2\tilde{M}_1\tilde{D} - k_5\left( \tilde{M}_{2c} - M_{2c}(0)\right) \tilde{D} - k_8\left( \tilde{M}_{2c}-M_{2c}(0)\right) \tilde{M}_{2a}, \end{aligned}$$

18 $$\begin{aligned} 0&= k_5\left( \tilde{M}_{2c}-M_{2c}(0)\right) \tilde{D} + k_6\tilde{M}_{2a}\tilde{M}_3 - k_7\tilde{M}_1\tilde{M}_{2a} + k_8\left( \tilde{M}_{2c}-M_{2c}(0)\right) \tilde{M}_{2a} \nonumber \\&\quad \ - k_9\left( \tilde{M}_{2a} - M_{2a}(0)\right) , \end{aligned}$$

19 $$\begin{aligned} 0&= k_3\tilde{M}_1\tilde{M}_{2c} + k_7\tilde{M}_1\tilde{M}_{2a} - k_4\tilde{M}_1\tilde{M}_3 - k_6\tilde{M}_{2a}\tilde{M}_3, \end{aligned}$$

20 $$\begin{aligned} 0&= a_1\tilde{M}_1\tilde{M}_b - a_2\left( \tilde{M}_b - M_b(0) \right) \tilde{M}_{2a}, \end{aligned}$$

21 $$\begin{aligned} 0&= a_2\left( \tilde{M}_b - M_b(0) \right) \tilde{M}_{2a} - a_4\tilde{M}_d - a_5\tilde{M}_1\tilde{M}_d - \dfrac{a_3\tilde{M}_d\tilde{F}}{\tilde{M}_{2c}-M_{2c}(0) + 1}. \end{aligned}$$

Consider Eq. (15) either $\tilde{D} = 0$ or the macrophage populations would be zero. If $\tilde{M}_1=0$, $\tilde{M}_{2a}=0$, and $\tilde{M}_{2c} = 0,$ then by Eq.  (16) we would have $\tilde{D}=0.$ Summing Eqs. (16)–(19) and using $\tilde{D}=0$, we have:

22 $$\begin{aligned} 0 = \dot{M}_{2a} = -k_9 \left( \tilde{M}_{2a} - M_{2a}(0) \right) \ne 0. \end{aligned}$$

Hence, we have a contradiction. This means that $\tilde{M}_1$, $\tilde{M}_{2a}$, and $\tilde{M}_{2c}$ cannot all be zero. Notice that Eq. (22) also implies that $\tilde{M}_{2a}\ne 0.$ Since $\tilde{M}_1$, $\tilde{M}_{2a}$, and $\tilde{M}_{2c}$ are nonnegative, $\tilde{D} = 0.$

Consider Eq. (17). Since $\tilde{D}=0$ and $\tilde{M}_{2a} > 0,$ we will have $\tilde{M}_{2c} = M_{2c}(0).$ Hence, by Eq. (13), $\tilde{R}=0.$ This leads to a dilema. Equation (12) combined with $\tilde{M}_{2c} = M_{2c}(0)$ and $\tilde{R}=0$ implies that either $\tilde{M}_d = 0$ or $\tilde{F} = 0$.

Assume first that $\tilde{M}_d = 0$, then by Eq.  (21) we have that $\tilde{M}_b = M_b(0) > 0.$ Thus, Eq. (20) yields $\tilde{M}_1 = 0$. Hence, by Eq.  (19) we have $\tilde{M}_3=0.$ Notice that Eqs. (12) and (14) are linearly dependent and give us a free variable, $\tilde{F}$. This give us:

23 $$\begin{aligned} \tilde{x} = \left[ \tilde{F},0,100-\tilde{F},0,0,M_{2c}(0),M_{2a}(0),0,M_b(0),0 \right] \end{aligned}$$

Assume now that $\tilde{F} = 0,$ then by Eq. (16) we have that either $\tilde{M}_1=0$ or $\tilde{M}_3 = \frac{k_3}{k_4}M_{2c}(0).$

If $\tilde{M}_1=0$, then $\tilde{M}_3 = 0$ from Eq.  (19). Also, $\tilde{M}_b = M_b(0)$ by Eq.  (20). Thus, $\tilde{M}_d = 0$ by Eq. (21). Notice, however, this gives you a fixed point of $\tilde{x_1} = [0,\ 0,\ 100,\ 0,\ 0,\ M_{2c}(0),\ M_{2a}(0),\ 0,\ M_b(0),\ 0]$ which is a special case of the earlier fixed point.

If $\tilde{M}_3 = \frac{k_3}{k_4}M_{2c}(0),$ then substituting into Eq. (18) we have:

$$\begin{aligned} \tilde{M}_1 = \dfrac{k_6\tilde{M}_{2a}\tilde{M}_3}{k_7\tilde{M}_{2a}} = \dfrac{k_3k_6M_{2c}(0)}{k_4k_7} \end{aligned}$$

However, by Eq. (20), this leads to:

$$\begin{aligned} M_b = \dfrac{-a_2M_b(0)M_{2a}(0)}{a_1\tilde{M}_1 - a_2M_{2a}(0)}. \end{aligned}$$

Using Eq. (21), we have:

$$\begin{aligned} \tilde{M}_d&= \dfrac{a_1a_2k_3k_6M_{2c}(0)M_{2a}(0)M_b(0)}{a_2k_4k_7M_{2a}(0) - a_1k_3k_6M_{2c}(0)} \cdot \dfrac{1}{ a_4 + a_5\tilde{M}_1} \\&= \dfrac{a_1a_2k_3k_4k_6k_7M_{2c}(0)M_{2a}(0)M_b(0)}{\Big (a_2k_4k_7M_{2a}(0) - a_1k_3k_6M_{2c}(0)\Big )\Big (a_4k_4k_7 + a_5k_3k_6M_{2c}(0) \Big )}. \end{aligned}$$

Therefore, we have that our fixed point as

$$\begin{aligned} \tilde{x}_2 = \left[ \begin{array}{c} 0, \\ 0, \\ 100, \\ 0, \\ \dfrac{k_3k_6M_{2c}(0)}{k_4k_7}, \\ M_{2a}(0), \\ M_{2c}(0), \\ \dfrac{k_3M_{2c}(0)}{k_4}, \\ \dfrac{a_2k_4k_7M_b(0)M_{2a}(0)}{a_2k_4k_7M_{2a}(0) - a_1k_3k_6M_{2c}(0)}, \\ \dfrac{a_1a_2k_3k_4k_6k_7M_{2c}(0)M_{2a}(0)M_b(0)}{\Big (a_2k_4k_7M_{2a}(0) - a_1k_3k_6M_{2c}(0)\Big )\Big (a_4k_4k_7 + a_5k_3k_6M_{2c}(0) \Big )} \end{array} \right] . \end{aligned}$$

$$\square $$

In the fixed point $\tilde{x}_1,$ we have a free variable $\tilde{F}$. This indicates that the associated steady state depends on when *F*(*t*) (the transient function of fibrous tissue) becomes constant. Since these values when near steady state—notice that regenerating tissue should already be close to zero—depend only on $M_d(t)$ (the transient function of differentiated myocytes), the FRiND model shows that the replacement of normal tissue by fibrous tissue depends on locally available differentiated myocytes. This observation is in agreement with Ogawa et al. (2015) and the model created by Virgilio et al. (2018). Normally, this is not an issue since myoblasts proliferate enough during the pro-inflammatory stage to give enough differentiated myocytes to repair muscle. However, this process can be disrupted enough for the free variable to become an issue as we will see in Result Section.

Notice that $\tilde{x}_2$ can violate assumption (2). **This would allow a model to approach a steady state that is unrealistic.** Stability analysis will show that the FRiND model will generally repel from this outcome. Theorem 2 will give a handy criteria for $\tilde{x}_2$ existing.

#### Theorem 2

If $a_1k_3k_6 M_{2c}(0) < a_2k_4k_7 M_{2a}(0),$ then $\tilde{M}_b$ and $\tilde{M}_d$ are nonnegative and the second steady state exists.

#### Proof

Let $a_1k_3k_6 M_{2c}(0) < a_2k_4k_7 M_{2a}(0).$ Notice that $a_2k_4k_7M_b(0)M_{2a}(0)$ is nonnegative. Thus, $\tilde{M}_b$ will be nonnegative if the denominator is positive. Since $a_1k_3k_6 M_{2c}(0) < a_2k_4k_7 M_{2a}(0)$, we know that $a_2k_4k_7 M_{2a}(0) - a_1k_3k_6 M_{2c}(0) > 0.$ Therefore, $\tilde{M}_b$ exists and is nonnegative. Furthermore, we have that

$$\begin{aligned} \tilde{M}_d = \dfrac{\overbrace{a_1a_2k_3k_4k_6k_7M_{2c}(0)M_{2a}(0)M_b(0)}^{\ge 0}}{\Big (\underbrace{a_2k_4k_7M_{2a}(0) - a_1k_3k_6M_{2c}(0)}_{> 0}\Big )\Big (\underbrace{a_4k_4k_7 + a_5k_3k_6M_{2c}(0)}_{> 0} \Big )}. \end{aligned}$$

Hence, $\tilde{M}_d$ exists and is nonnegative.

Therefore, all components of the second fixed point exist and are nonnegative. The FRiND model will have a second steady state. $\square$

The FRiND model with initial conditions (Table 1) and parameters (Table 2) will fail the above criteria. Therefore, the only fixed point we need to worry about is:

$$\begin{aligned} \tilde{x}_1 = [F,0,100-F,0,0,200,200,0,100,0]. \end{aligned}$$

### Stability Analysis

A steady state can be called stable, unstable, or saddle depending on the system’s reaction to small perturbations to the fixed point **(other types of reactions can occur but will not be needed in this discussion)**. The system will return to a steady state after perturbations when a fixed point is stable, whereas the system will repel if the fixed point is unstable. With a saddle fixed point, the system will repel from the point unless on specific vectors (Strogatz 2018).

The sufficient conditions for stability are given by eigenvalues of the Jacobian evaluated at the fixed points. A stable steady state will have only **real, nonpositive** eigenvalues. An unstable state will have only **real, nonnegative** eigenvalues, and a saddle steady state will have a mix of positive and negative, **real** eigenvalues (Strogatz 2018).

The eigenvalues of the Jacobian evaluated at $\tilde{x}_1$ are

$$\begin{aligned} \lambda _1 = \left[ \begin{array}{c} 0 \\ 0 \\ 0 \\ -k_9 \\ -M_{2a}(0) k_8 \\ -k_6 M_{2a}(0) \\ -k_3M_{2c}(0) \\ -d_{4}M_{2a}(0){-}d_5M_{2c}(0) \\ -d_2M_{2a}(0){-}d_1M_{2c}(0) \\ -a_4{-}a_5 \end{array} \right] . \end{aligned}$$

**According to the assumptions listed earlier, the parameters and initial values are positive. Thus,** the eigenvalues are **real and** nonpositive. Therefore, the steady state at $\tilde{x}_1$ is stable.

The eigenvalues of the Jacobian evaluated at $\tilde{x}_2$ are

$$\begin{aligned} \lambda _2 = \left[ \begin{array}{c} 0\\ -k_9\\ -M_{2a}(0)k_8\\ -1/2\frac{M_{2a}(0)k_6k_7+M_{2c}(0)k_3k_6-\sqrt{M_{2a}(0)^2k_6^2k_7^2+2M_{2a}(0)M_{2c}(0)k_3k_6^2k_7+4k_7^2M_{2a}(0)M_{2c}(0)k_3k_6+M_{2c}(0)^2k_3^2k_6^2}}{k_7}\\ -1/2\frac{M_{2a}(0)k_6k_7+M_{2c}(0)k_3k_6+\sqrt{M_{2a}(0)^2k_6^2k_7^2+2M_{2a}(0)M_{2c}(0)k_3k_6^2k_7+4k_7^2M_{2a}(0)M_{2c}(0)k_3k_6+M_{2c}(0)^2k_3^2k_6^2}}{k_7}\\ -\frac{M_{2a}(0)d_4k_4k_7+M_{2c}(0)d_3k_3k_6+M_{2c}(0)d_5k_4k_7}{k_4k_7}\\ -M_{2a}(0)d_2-M_{2c}(0)d_1\\ -\frac{M_{2c}(0)k_3k_6+a_4k_4k_7+a_5k_4k_7}{k_4k_7}\\ \frac{M_{2c}(0)a_1k_3k_6}{k_4k_7}\\ -\frac{M_{2a}(0)M_b(0)M_{2c}(0)a_1a_2d_6k_3k_4k_6k_7}{M_{2a}(0)M_{2c}(0)a_2a_5k_3k_4k_6k_7-M_{2c}(0)^2a_1a_5k_3^2k_6^2+M_{2a}(0)a_2a_4k_4^2k_7^2-M_{2c}(0)a_1a_4k_3k_4k_6k_7} \end{array}\right] . \end{aligned}$$

**Again by the earlier assumptions,**$M_{2c}(0),\ a_1,\ k_3,\ k_6,\ k_4,$**and**$k_7$**are positive.Hence, all the eigenvalues are real, and** the eigenvalue $\frac{M_{2c}(0)a_1k_3k_6}{k_4k_7}$**is always positive** and $-k_9$ is always negative. Therefore, the steady state at $\tilde{x}_2$ is a saddle node.

The **above** stability analysis shows that the FRiND model will **converge to**$\tilde{x}_1$ unless on specific vectors **(which will converge to**$\tilde{x}_2$). The flexibility given by the free variable in $\tilde{x}_1$ will allow for the comparison of the normal steady state for the immune system to steady states that include replacement of normal muscle fiber with fibrous connective tissue.

### Identifiability and Sensitivity Analysis

The question could arise at this point whether the FRiND model’s parameters can be estimated by any appropriate dataset. In mathematics, this concept is called identifiability. Models which are unidentifiable can be reduced by eliminating parameters; this helps to estimate parameters faster and creates a uniqueness to parameter estimation. This concept is related to sensitivity analysis. A system which is highly sensitive will be at least locally identifiable (Eisenberg and Hayashi 2014; Fisher 1959).

To calculate the sensitivity matrix numerically, we estimated $\dfrac{{\partial }x_k}{{\partial }p_j}(t_i)$ for each *i*, *j*, *k* with the central-difference formula. We also **tried** more accurate estimations of **the partial** derivatives before writing this article but found the results to be similar. We calculated the sensitivity matrix and Fisher Information Matrix (FIM) as described in Eisenberg and Hayashi (2014).

The **eigenvalues of the** FIM for the FRiND model were

$$\begin{aligned} Eig_{FIM} =\&[0.04,&21.46,&3.43e3,&3.33e5,&6.99e5,&2.90e7,&2.28e9,&\ldots \\&9.41e9,&3.04e10,&6.01e10,&1.34e11,&4.72e11,&7.01e11,&9.08e12,&\ldots \\&2.76e13,&2.93e14,&7.85e14&1.61e15,&2.39e16,&1.55e18,&9.23e22 ].&\end{aligned}$$

**This gives us the determinant of the FIM matrix as**$1.0422 \cdot 10^{226}.$ As the determinant is large, the parameters of the FRiND model are sensitive and at least locally identifiable (Eisenberg and Hayashi 2014; Fisher 1959).

## Results

One of the goals of the FRiND model is to use a damage function, *f*(*t*), to study the effects of damage to muscle tissue from chronic immune activation; specifically, the FRiND model allows exploration into how the acute immune response becomes a chronic immune response which causes muscle to be replaced by fibrous tissue.

To accomplish this goal, **we must show that that model is accurate on the given data used to estimate the model parameters** (Sect. 2.3) which were obtained from an acute damage event. After the accuracy of the model for given data has been shown, we will use the FRiND model to predict other experiments and results for which the model has not been trained including simulations of chronic muscle damage and effects of diphtheria toxin on muscle repair. All time-course graphs were created using Adams–Bashforth–Moulton Predictor–Corrector method (Cheney and Kincaid 2007) on MATLAB and verified using COPASI (which uses LSODA) (Hoops et al. 2006).

### The FRiND Model is Consistent with the Data in Table 1

As mentioned above, parameters were estimated using data from the literature (Mounier et al. 2013; Ogawa et al. 2015) (Table 1). The parameter values are reported in Table 2. As discussed in Sect. 2.3, we chose the **Gaussian** function (Eq. (11)) to simulate damage from Cardiotoxin (CTX) (Dell’Acqua and Castiglione 2009; Jarrah et al. 2014).

**Fig. 4:**
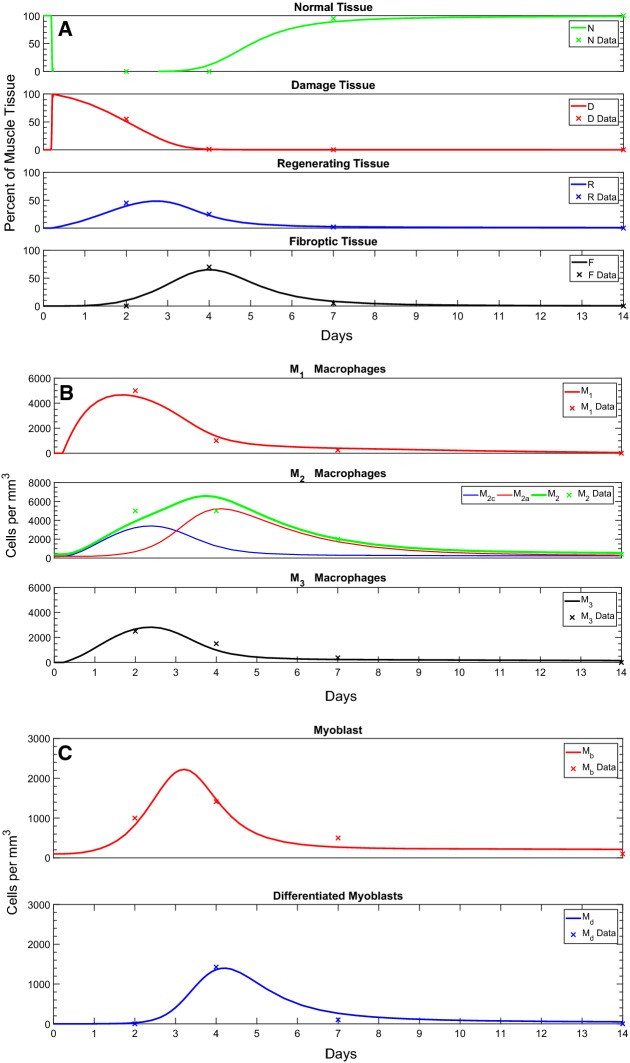
*Time course of FRiND model* These figures show the time course of the **a** muscle tissue equations, **b** macrophage equations, and **c** myocytes equations. Actual data in Table 1 are given as *x*’s on the graphs (Color figure online)

**Fig. 5:**
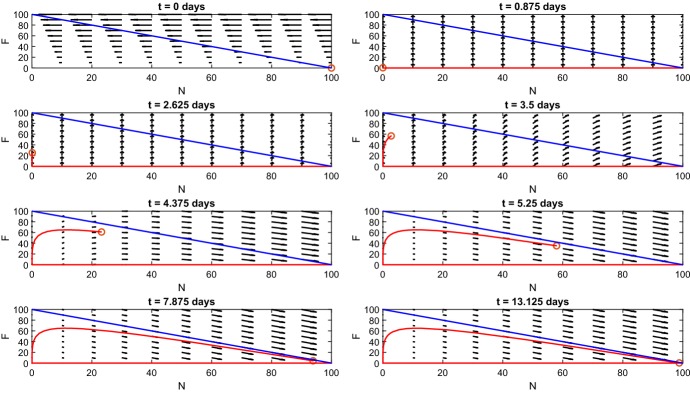
*Phase portrait of*$N \times F$*plane.* This figure shows the time course in the $N \times F$ phase plane, where vector arrows show the direction of flow and the red circle gives position of $\left( N(t),F(t)\right) .$ The blue line $F = N - 100$ acts as a barrier where the red circle cannot cross since $N(t) + F(t) = 100$ (Color figure online)

**Fig. 6:**
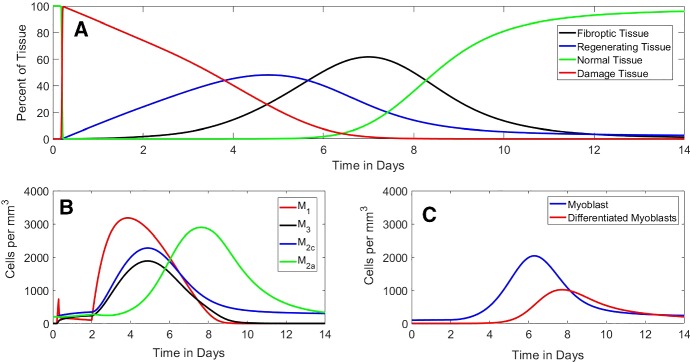
*Diphtheria-treated mice.* These figures show the time course for the FRiND model with $M_1$ macrophages depleted by DT. The results are consistent with Arnold et al. (2007). **a** shows the results for muscle tissue. **b** shows the results for macrophages. **c** shows the time course of myocytes (Color figure online)

Muscle tissue predictions followed closely to that given by Mounier et al. (2013). From Fig. 4a, normal tissue experiences a steep drop as muscle is exposed to CTX; normal tissue is completely compromised within 24 h. Damage peaks within the first 24 h followed by complete phagocytosis over the next 3 days. The model gives regenerating tissue at about $40\%$ at day 2 which is slightly less than phagocyted myofibers given in the literature (Mounier et al. 2013). However, the model’s values for regenerating tissue at days 4 and 7 are closer. Fibrous tissue occurs earlier and peaks slightly lower than that reported by the literature (Mounier et al. 2013). Recovery of new normal tissue does occur within the reported period (Fig. 5). The model also closely follows the trends in tissue data reported by Arnold et al. (2007) which used a notexin (Fig. 6).

Macrophage predictions followed the general characteristic of the data given in Mounier et al. (2013). From Fig. 4b $M_1$, macrophages peak between day 1 and day 2 as reported by Mounier et al. (2013); Arnold et al. (2007). However, the number of $M_1$ macrophages is less than expected. $M_2$ macrophages are noted to peak between days 2 and 4 as expected (Mounier et al. 2013; Arnold et al. 2007); the numbers do, however, understate day 2 and overshoot day 4 numbers. $M_3$ macrophage predictions closely follow data from Mounier et al. (2013).

Myocyte predictions closely follow the expected cell numbers before day 7 (Fig. 4c). After day 7, myoblast numbers fall short of expected numbers and differentiated myoblast numbers overshoot reported cell numbers (Ogawa et al. 2015).

### The FRiND Model is Consistent with Inflammatory Macrophage Inhibition by Diphtheria Toxin

Arnold et al. (2007) performed several experiments studying the actions of macrophages on muscle regeneration. The group noticed when $M_1$ macrophages are depleted within the first day after damage by the exposure to diphtheria toxin (DT) that necrotic (damaged) tissue removal is delayed significantly. Furthermore, the regeneration process resumes after $M_1$ macrophages were allowed to return and the steady state lacked significant replacement of normal tissue by fibrous tissue.

To simulate this, the FRiND model can be modified by subtracting $g(t)M_1$ from the $M_1$ macrophage equation. Thus,

$$\begin{aligned} \dot{M_1} = k_1D^2 - k_2M_1D - k_3M_1M_{2c} + k_4M_1M_3 - g(t)M_1, \end{aligned}$$

where

$$\begin{aligned} g(t) = \left\{ \begin{array}{rcl} 50 &{} \text{ for } &{} 0.3< t < 2 \\ 0 &{} \text{ for } &{} \text{ otherwise } \\ \end{array} \right. . \end{aligned}$$

Figure 6 shows the results of this adapted FRiND model. $M_1$ macrophages are depleted shortly after diphtheria toxin is administered and rebounds around day 2 when $k(t) = 0.$ While $M_1$ macrophages are depleted, only a small amount of necrotic tissue is removed mainly by $M_2$ macrophages already in the tissue. The repair process resumes after DT subsides and $M_1$ macrophage numbers rebounds with full recovery by 14 days. The steady state of the system also shows that no significant fibrosis remains by 14 days. This matches the results given by Arnold et al. (2007).

### The FRiND Model is Consistent with Chronic Immune Activation and Literature Results in *mdx* Mice

The archetypal pathology of MD is the replacement of healthy muscle tissue with fibrous tissue caused by severe acute damage to muscle from normal daily activity (Desguerre et al. 2009). Most studies of MD and muscle repair revolve around performing a single acute muscle damage. However, MD patients/mice rarely suffer from just a single damage event; instead normal daily activities cause both mild and severe acute events throughout the body. We would like to use the FRiND model to predict this condition by only changing the input damage function. This **could** show that repeat**ed** severe acute damage events cause the chronic immune response and replacement of muscle tissue**, which precipitates the muscle weakness in MD.**

A choice for this daily damage could be $f(t) = a \sin {(bt+c)}$ for some choice of parameters *a*, *b*, *c*. However, to avoid negative damage we should use $f(t) = a \sin ^2{(bt+c)}.$**Since we can dictate whether**$t=0$**is at midnight or midday, without loss of generality, we can choose**$b = \pi$ and $c=0$ which would give daily periodic damage peaking at midday or midnight. **To simulate normal daily muscle usage, this model will allow***a***to be a random number between** 0 **and** 1 **which will be selected each day.For** Theorem 1**to apply in the FRiND model, damage added by***f***must either converge to zero naturally or by setting the function to zero at some time point.**

The following results are again given using Adams–Bashforth–Moulton Predictor–Corrector method on MATLAB and $f(t) = a \sin ^2{(\pi t)}$ .

**Fig. 7:**
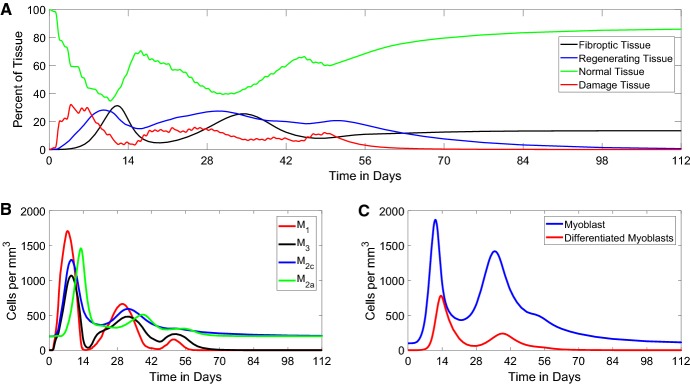
*Non-fading periodic damage.* These figures show the time course for the FRiND model with $f(t) = a \sin ^2{(\pi t)}$ with *a* randomly selected each day. **a** The results for muscle tissue. The steady state of fibrous tissue (*F*) is close to the experimental outcomes of Lu et al. (2014) which found a $20\%$ fibrous replacement for 8-week-old *mdx*/utrn$^{-/-}$ mice. **b** The results for macrophages. **c** The time course of myocytes (Color figure online)

Figure 7 shows that over a period of several weeks, normal tissue is repaired with some replacement by fibrous tissue. $M_1$ macrophages are nullified by $M_{2c}$ macrophages and prevented from encouraging proliferation of more myoblasts. This causes a lack of differentiated myoblasts to bind with fibrous tissue into normal tissue; a buildup of fibrous tissue follows. Normal tissue is partially replaced by fibrous tissue around 16 weeks. A partial replacement by fibrous tissue matches studies of gastrocnemius muscle in *mdx*/utrn$^{-/-}$ mice (Lu et al. 2014) **which found around 20% of normal tissue was replaced by collagen-positive tissue after 8 weeks**.

**Fig. 8:**
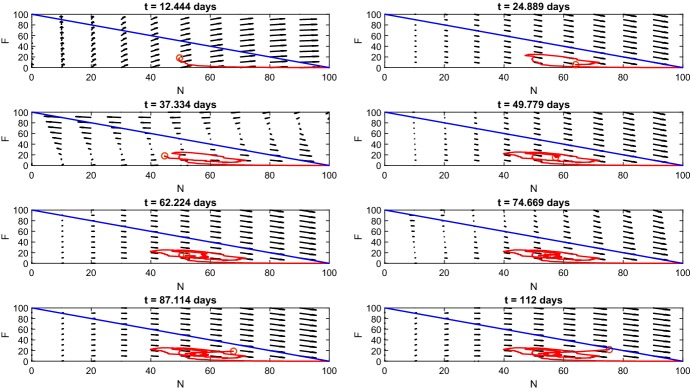
*Phase portrait of*$N \times F$*plane for non-fading periodic damage.* This figure shows the time course in the $N \times F$ phase plane where vector arrows show the direction of flow and the red circle gives the position of $\left( N(t),F(t)\right) .$ The blue line $F(t) = N(t) - 100$ acts as a barrier where the red circle cannot cross since $N(t) + F(t) \le 100$ (Color figure online)

The phase portrait of the FRiND model (Fig.  8) when $f(t) = a \sin ^2{(\pi t)}$ emphasizes the cyclic nature of the process. Fibrous and normal tissue interchange constantly creating jagged ellipses throughout the first 11 weeks. Following the depletion of differentiated myoblasts, the phase portrait shows that the cyclic nature ends and spirals toward the $F(t) = N(t) - 100$ line which is a push toward a steady state with elevated fibrous tissue and loss of normal tissue (both damage and regenerating tissue **will approach** zero by the 16*th* week.

**Fig. 9:**
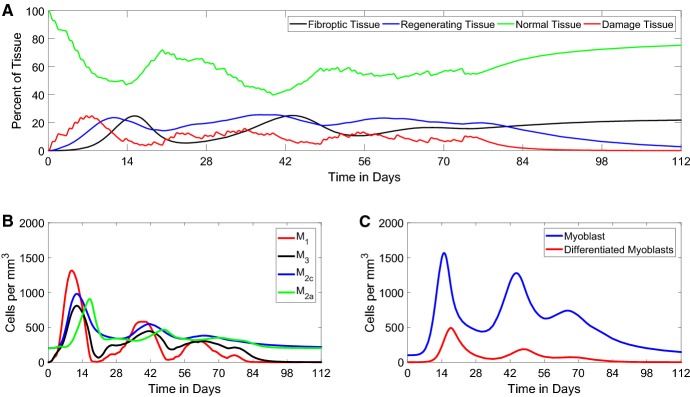
*Fading periodic damage.* These figures show the time course for the FRiND model with $f(t) = \frac{a\sin ^2{(\pi t)}}{t+1}.$**a** The results for muscle tissue. The steady state of fibrous tissue (*F*) is close to the experimental outcomes of Salimena et al. (2000) which found 4%/7% (male/female) and Sun et al. (2015) which found $7\%$ in *mdx* mice. **b** shows the results for macrophages. **c** The time course of myocytes (Color figure online)

**Fig. 10:**
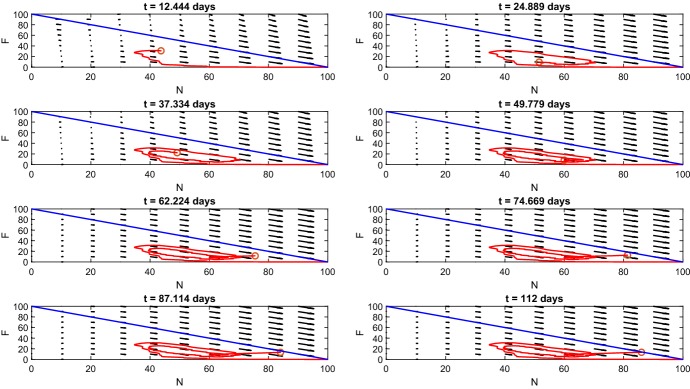
*Phase portrait of*$N \times F$*plane for fading periodic damage.* This figure shows the time course in the $N \times F$ phase plane where vector arrows show the direction of flow and the red circle gives the position of $\left( N(t),F(t)\right) .$ The blue line $F(t) = N(t) - 100$ acts as a barrier where the red circle cannot cross since $N(t) + F(t) = 100$ (Color figure online)

A similar function to consider is $f(t) = \frac{a\sin ^2{(\pi t)}}{t+1}$; this function allows for weakening of a patient’s strength over time as muscle is replaced by fibrous tissue. Using this damage function, we can create results (Figs.  9 and 10) with similar properties as the earlier non-fading damage function and fibrous tissue **reaching steady state** around $7\%$ which closely aligns with *mdx* mice studies **which found 4%/7% (male/female)** (Salimena et al. 2000) **and 7%** (Sun et al. 2015).

Further studies of MD can be gleamed from the FRiND model without changing *f*.

## Discussion

All forms of MD are rare (Norwood 2009), and the future of MD research might rely on mathematical modeling when insufficient test subjects are available. However, few mathematical models have been created to quantify the experiments about MD (Houston et al. 2018).

Although most forms of MD are genetic disorders, chronic inflammation caused by repeated severe acute immune responses has been implicated in several forms of MD (Wehling et al. 2001; Selva-O’Callaghan et al. 2006; Spencer et al. 2001; Desguerre et al. 2009; Tidball and Villalta 2010). The two previous models (Dell’Acqua and Castiglione 2009; Jarrah et al. 2014) attempted to simulate research of inflammation during the lifetime of *mdx* mice (Wehling et al. 2001; Spencer et al. 2001). Both models used the law of mass action with three immune cells: CD8+ and CD4+ T cells and pro-inflammatory macrophages. They used a single acute damage event to start a perturbation that estimated chronic inflammation. The models were also bistable; either the normal tissue completely returned or a little damage remained permanently.

However, the previous models miss several key aspects of tissue repair. Treating macrophages as a homogeneous group reduces the predictive power of those models. Macrophages display several phenotypes that perform different tasks during muscle repair; some of which play an important role in laying fibrous connective tissue and migrating nascent myotubes to form new muscle; previous models handled this by using linear mass action dynamics for tissue repair after damaged tissue was eaten by pro-inflammatory macrophages. We introduced the FRiND model to rectify these issues.

The FRiND model offers many avenues for future usage by integrating macrophage plasticity and nonlinear mass action dynamics. The ability to adapt to experiments with different criteria for subject injury and to study different pharmaceutical outcomes by changing parameters in the system could be useful in exploring biological outcomes in muscle regeneration.

We have shown that changing *f*(*t*) in the FRiND model can be used to explore outcomes that depend on the injury caused to muscle tissue. In addition, this injury can be different than the injury used to give the estimating parameters. The parameters used throughout the results section were estimated using control mice after CTX damage. The results studied, however, were free to change the *f*(*t*) to explore other forms of muscle damage like normal degradation due to muscular dystrophy. This could hint that the weakening of skeletal muscle in several types of MD is the result of the immune system’s overreaction due to compromised muscle cell structure.

The FRiND model also predicts experimental and pharmaceutical outcomes by changing or adding parameters into the model. As shown in Sect. 3.2, an added parameter in the model exhibits the same general trends reported by Arnold et al. (2007). This could allow future outcomes to be tested and studied before performing an experiment.

The FRiND model does include some simplifications. The model does not include several immune and non-immune cells which have been shown to be significant in tissue repair including neutrophils (Arnold et al. 2007), T cells (Tidball and Villalta 2010; Burzyn et al. 2013), fibro/adipogenic progenitors (Joe et al. 2010), and tissue cells under inflammation. With more data, many of these cells could be incorporated into the FRiND model. Inclusion of these cells and many others could allow further elucidation into the repair process.

The FRiND model also simplifies spatial dynamics by following the law of generalized mass action which assumes interactions are isotropic. Future research could expand this system into an anisotropic model using partial differential equations (PDEs). This could also allow a three-dimensional study of muscle regeneration. Another direction could include stochastic differential equations to differentiate early repair when cells are first infiltrating from later **stages when cells are nearly isotropic.**
